# Neutrophils Contribute to the Protection Conferred by ArtinM against Intracellular Pathogens: A Study on *Leishmania major*

**DOI:** 10.1371/journal.pntd.0004609

**Published:** 2016-04-08

**Authors:** Rafael Ricci-Azevedo, Aline Ferreira Oliveira, Marina C. A. V. Conrado, Fernanda Caroline Carvalho, Maria Cristina Roque-Barreira

**Affiliations:** Departamento de Biologia Celular e Molecular e Bioagentes Patogênicos, Faculdade de Medicina de Ribeirão Preto, Universidade de São Paulo, Ribeirão Preto, São Paulo, Brasil; FIOCRUZ - Minas, BRAZIL

## Abstract

ArtinM, a D-mannose binding lectin from *Artocarpus heterophyllus*, has immunomodulatory activities through its interaction with N-glycans of immune cells, culminating with the establishment of T helper type 1 (Th1) immunity. This interaction protects mice against intracellular pathogens, including *Leishmania major* and *Leishmania amazonensis*. ArtinM induces neutrophils activation, which is known to account for both resistance to pathogens and host tissue injury. Although exacerbated inflammation was not observed in ArtinM-treated animals, assessment of neutrophil responses to ArtinM is required to envisage its possible application to design a novel immunomodulatory agent based on carbohydrate recognition. Herein, we focus on the mechanisms through which neutrophils contribute to ArtinM-induced protection against *Leishmania*, without exacerbating inflammation. For this purpose, human neutrophils treated with ArtinM and infected with *Leishmania major* were analyzed together with untreated and uninfected controls, based on their ability to eliminate the parasite, release cytokines, degranulate, produce reactive oxygen species (ROS), form neutrophil extracellular traps (NETs) and change life span. We demonstrate that ArtinM-stimulated neutrophils enhanced *L*. *major* clearance and at least duplicated tumor necrosis factor (TNF) and interleukin-1beta (IL-1β) release; otherwise, transforming growth factor-beta (TGF-β) production was reduced by half. Furthermore, ROS production and cell degranulation were augmented. The life span of ArtinM-stimulated neutrophils decreased and they did not form NETs when infected with *L*. *major*. We postulate that the enhanced leishmanicidal ability of ArtinM-stimulated neutrophils is due to augmented release of inflammatory cytokines, ROS production, and cell degranulation, whereas host tissue integrity is favored by their shortened life span and the absence of NET formation. Our results reinforce the idea that ArtinM may be considered an appropriate molecular template for the construction of an efficient anti-infective agent.

## Author Summary

Vaccination is a successful way to eliminate infectious diseases. The generated antibodies neutralize the invading microbe and avoid the establishment of infection. Vaccines are efficient to prevent infections by pathogens living outside rather than inside the host`s cells. This occurs because protection against intracellular pathogens requires the engagement of T lymphocytes. The discovery of receptors on innate immunity cells opened new perspectives in trying manners to stimulate effective response against intracellular pathogens. Frequently the microbial sensing by Toll-like receptors (TLRs) besides triggering immediate defense also orchestrates adaptative immunity towards T-cell response. Therefore, TLR ligands started to be assayed in new anti-infective approaches. Our laboratory has been investigating the immunomodulation induced by lectins, which are ubiquitous sugar-binding proteins. Our primary model is ArtinM, from the seeds of jackfruit, a lectin that binds to TLR2 sugar chains on macrophages and dendritic cells and promotes production of cytokines that engages T lymphocytes in a process that culminate with elimination of intracellular pathogens. Concomitantly, ArtinM activates other immune cells, including neutrophils, which contributes to the pathogen elimination, but may also account for tissue damage. This last possibility led us to investigate the lectin effects on neutrophils deeply. We analyzed neutrophils treated with ArtinM and infected with *Leishmania major*. We concluded that the leishmanicidal ability of ArtinM-stimulated neutrophils was due to augmented release of inflammatory cytokines, ROS production, and cell degranulation. Otherwise, host tissue integrity is favored by shortened cells lifespan and absence of NET formation. This work reinforces the idea that ArtinM can be an appropriate molecular template for the construction of an efficient anti-infective agent.

## Introduction

Global immunization regimes have eradicated smallpox and controlled a large number of other infections [1,2]. Indeed, vaccines have been successful against infections caused by extracellular pathogens or those whose pathogenesis is mediated by toxins. Under these circumstances, vaccines confer protection by inducing antibodies that neutralize the inoculum and prevent the establishment of infections [3]. This effective formula is not applied to prevent infections with intracellular pathogens [4] because they require T-cell mediated immunity for elimination. Therefore, a research field has emerged that concerns the development of alternative prophylactic or therapeutic agents to enhance host cellular response. In this context, agonists of innate immunity receptors, especially Toll-like receptors (TLRs), provide promising approaches [5]. The interaction of agonists with TLRs triggers cell signaling and production of inflammatory and anti-inflammatory mediators [6]. This process, beyond inducing early mechanisms of host defense, primes and orchestrates antigen-specific adaptive responses [7]. The ability of activated TLRs to modulate adaptive immunity motivates ongoing trials of new drugs based on natural or synthetic TLR ligands for infectious diseases in humans [8].

ArtinM, a D-mannose-binding lectin obtained from the seeds of *Artocarpus heterophyllus*, binds to TLR2 N-glycans and functions as a TLR2 agonist that exerts immunomodulatory properties [9]. The ArtinM interaction with TLR2 on macrophages and dendritic cells results in high levels of IL-12 production, driving immunity towards the T helper (Th) 1 axis [10]. This ability accounts for the protection conferred by ArtinM administration against *Leishmania major* [11], *Leishmania amazonensis* [12], *Paracoccidioides brasiliensis* [10,13], *Neospora caninum* [14], and *Candida albicans* [15] infections in mice. Beyond acting on antigen presenting cells, ArtinM exerts activities on lymphocytes [16], mast cells [17,18], and neutrophils [19,20]. This pleiotropic activity on immune cells is considered to account for the ArtinM property of conferring resistance against intracellular pathogens [21].

Concerning neutrophils, the cell type focused in this work, they are known to participate in the protection against intracellular pathogens, through mechanisms that involve phagocytosis, cell degranulation, ROS production, release of lipid mediators, and formation of neutrophil extracellular traps (NETs) [22]. Further mechanisms known to favor host defense are the release of cytokines combined with changes in cell life span [23]. Our previous work showed that ArtinM induces neutrophil migration by haptotaxis [24], due to the concomitant interactions of ArtinM CRDs with N-glycans on neutrophil surface receptors, such as those linked to C-X-C chemokine receptor 2 (CXCR2), and glycoproteins of the extracellular matrix, such as laminin [25,26]. Also, ArtinM activates neutrophils, causing tyrosine phosphorylation, L-selectin shedding, and interleukin-8 (IL-8) and leukotriene B4 (LTB4) secretion. These responses result in the enhancement of phagocytic and microbicidal abilities of neutrophils [19,20] and indicate that ArtinM activates neutrophils hugely.

Although effective against pathogens, neutrophils also account for exacerbated inflammation and tissue injury [27], a fact that caused concerns regarding the possibility of using ArtinM to design a novel class of immunomodulatory agents acting through carbohydrate recognition. Although exacerbated inflammation was never observed in the ArtinM-treated animals, we always had concerns regarding the occurrence of inflammatory tissue injury. Indeed, it is unclear how ArtinM may take advantage of neutrophil activation to eliminate pathogens, without promoting tissue damage. Therefore, in this study, we focused on understanding the mechanisms through which neutrophils contribute to the protective effect of ArtinM against intracellular pathogens and how this process occurs without exacerbating inflammation.

## Materials and Methods

### Ethics statement

The Ethics Committee of the Clinical Hospital of the Faculty of Medicine of Ribeirão Preto, University of São Paulo, approved this study (Doc. Number: 10012/2009) and all the adult volunteers signed an informed consent form prior to blood and/or urine donation.

### ArtinM preparations and treatment

ArtinM (ID: Q7M1T4_ARTIN, available on UniProtKB database) was extracted from *Artocarpus heterophyllus* seeds and purified by sugar affinity chromatography as previously described by Santos-de-Oliveira et al. (1994) [24]. The concentration of ArtinM lectin used to treat the neutrophils in this study was 2.5 μg/mL, as previously utilized [20], except in some assays, as specified.

### Neutrophil isolation

Heparinized human blood was layered on a density gradient of a neutrophil isolation medium (Monopoly Resolving Medium; ICN Pharmaceuticals, USA), as previously described by Toledo et al. (2009) [20]. The collected polymorphonuclear (PMN) cells were washed twice; a fraction was labeled with anti-CD16b antibody (BD—Biosciences, USA), and the purity was analyzed by flow cytometry (<95%, S1A Fig), and additionally by cytology from cytocentrifuged preparations Diff-Quick-stained (Laborclin, Brazil) (S1B Fig).

### *Leishmania major* culture and infection

*L*. *major* (LV39 strain) promastigotes were grown in Schneider’s medium supplemented with 1% antibiotic-antimycotic, 10% heat-inactivated fetal calf serum (FCS), 2 mM L-glutamine (all from Invitrogen, USA), and 2% human urine, pH 7.2. Neutrophils were pre-treated with ArtinM, and after 30 min, they were infected with stationary-phase promastigotes (5–7 day) at a parasite-PMN ratio of 3:1. Control neutrophils were not treated with ArtinM and not infected. Plates/tubes were centrifuged (300 × *g*/ 10 min) and incubated at 37°C in RPMI 1640 or Hank’s Balanced Salt Solution (HBSS) in a humidified atmosphere containing 5% CO_2_. Some assays were performed by infecting neutrophils with green-fluorescent *L*. *major* forms (mβT3-LV39), which were kindly provided by Prof. Angela Kaysel Cruz and Dr. Mônica Cristina Terrão.

### *Leishmania major* uptake by neutrophils

Human neutrophils (2 × 10^6^ cells/mL) were previously treated with ArtinM for 30 min, and infected with *L*. *major*. Infected/untreated neutrophils were used as controls. After 3 and 20 h post-infection, the cultures were cytocentrifuged (50 × g, 5 min) and Diff-Quik-stained (Laborclin, Brazil). The number of neutrophils with intracellular parasites was determined by count among 200 cells/condition/time, using an Olympus BX50 microscope coupled to a Nikon DXM-1200 photographic system.

### Parasite viability

To analyze the intracellular leishmanicidal activity, we assessed the parasite viability, as described by Tavares et al. (2014) [28], with slight modifications. Briefly, neutrophils that were treated with ArtinM and untreated controls were infected and cultured for 3 h with the parasites. Uninternalized parasites were discarded from cultures by washing twice for 5 min at 100 × *g*, and the culture was immediately fed with supplemented Schneider’s medium. The cells were then cultured at 26°C for an additional 48 h. The intracellular leishmanicidal activity was determined by assessing the number of extracellular motile promastigotes produced.

To analyze if some degranulated content had leishmanicidal activity, we performed a free cell assay based on methodology previously demonstrated by Mikus and Steverding (2000) [29]. Neutrophils treated with ArtinM for 1 h and untreated controls were pelleted, and the supernatants of the cells were collected. The supernatants were incubated with antibodies to myeloperoxidase (anti-MPO; ab62141; Abcam; 1/100) or neutrophil elastase (anti-NE; ab21595; Abcam; 1/100) for 30 min. Control supernatants were not incubated with the antibodies. After the incubation, 1 × 10^5^ parasites were added to the supernatants for 1 h, and then assayed for viability in Schneider’s medium containing Alamar Blue (Life Technologies, USA; 1/10 dilution) for an additional incubation of 24 h. Fluorescence measurement was performed on a FLx800 Fluorescence Microplate Reader (BioTek Instruments, USA; excitation, 590 nm; emission, 635 nm). Fluorescence count data from unchallenged, serially diluted parasites were used to obtain a standard curve of viable parasites.

### ELISA

Freshly isolated human neutrophils (2 × 10^6^ cells/mL) were previously treated with ArtinM for 30 min and infected with *L*. *major*. Neutrophils without ArtinM treatment and uninfected with *L*. *major* were used as negative controls. PMA stimulated (50 nM) neutrophils were used as the positive control. Cytokine levels were quantified in the neutrophil supernatants 20 h after infection and at the same time in the supernatants of controls. TNF, IL-1β (BD—Biosciences, USA), and TGFβ-1 (R&D Systems, USA) were measured by sandwich ELISA, according to the manufacturer’s instructions.

### Measurement of ROS production

The neutrophil chemiluminescence assay was performed in 96-well microplates using the procedure described by Lucisano-Valim et al. (2002) [30], with slight modifications. Freshly isolated human PMNs (2 × 10^5^ cells/well in HBSS) were mixed with the chemiluminescent probes luminol (0.1 mM) or lucigenin (0.1 mM). The mixture was incubated at 37°C for 3 min, and the reaction was initiated after adding ArtinM, phorbol myristate acetate (PMA, 0.1 μM), formyl-Met-Leu-Phe (fMLP, 1 μM), or HBSS. Some assays were performed by first incubating the cells with ArtinM during 30 minutes, and then by adding fMLP, PMA or *L*. *major* (MOI 3:1). The luminol- and lucigenin-enhanced chemiluminescence was measured in a microplate luminometer (LB 960 Centro, Berthold Technologies, Germany), and light emission was recorded in photon counts per second (CPS) for 30–60 min, at 37°C. AUC represents the area under the time–course curve, which was used to determine the total amount of measured ROS.

### Evaluation of Neutrophil Elastase (NE) activity

Neutrophil elastase activity was evaluated as previously described [31], with some modifications. Briefly, 1 × 10^5^ neutrophils were treated for 30 min with different concentrations of ArtinM (2.5 μg/mL to 312 ng/mL), fMLP (1 μM), or medium only, and NE activity was detected by using the substrate N-succinyl-alanine-alanine-valine-p-nitroanilide (SAAVNA) (1 mM), which is cleaved by the enzyme released in the supernatant, forming p-nitroaniline as one of the products, which was spectrophotometrically quantified, using the microplate reader PowerWaveX (BioTek Instruments, USA; 405 nm).

### Intracellular Myeloperoxidase (MPO) detection

Myeloperoxidase, as well as elastase, are azurophilic granule contents of neutrophils. Their intracellular levels were measured in neutrophils after 20 h of incubation with or without ArtinM, and challenged or not with *L*. *major*. To detach ArtinM-treated neutrophils, it was necessary to use EDTA-glucose (10 μM/5 μM) containing PBS (pH 7.2). Then, the cells were washed twice with PBS, and immediately fixed and permeabilized using the Cytofix/Cytoperm kit (BD—Biosciences, USA), following the manufacturer’s instructions. Next, the cells were incubated with anti-MPO^PE^ or anti-Hamster IgG1^PE^ isotype control antibodies (BD—Biosciences, USA), for 20 min. Immunofluorescent staining was analyzed by flow cytometry using Guava EasyCyte Mini (Millipore, USA).

### NET-DNA release measurement

Neutrophils (2 × 10^5^ cells/well in HBSS) were incubated with or without ArtinM, PMA (50 nM), or both together (ArtinM+PMA) in a 96-well plate for 2 and 4 h at 37°C, in 5% CO_2_. NET-DNA was quantified using a modified version of a previously published method [32]. Bacterial endonuclease *EcoRI* (200 unit/mL) was added for 10 min to partially digest any released NETs. Next, the cells and debris were pelleted by centrifugation at 600 × *g* for 10 min. A sample of the supernatant was added to SYTOX green nucleic acid stain (Invitrogen; 1 μM) in a black 96-well plate and incubated at 37°C for 10 min. NET-DNA fragments were quantified using a FLx800 Fluorescence Microplate Reader (BioTek Instruments, USA; excitation 485 nm, emission 528 nm) and the results have been expressed as Δ fluorescence values.

### NET immunofluorescence detection

Neutrophils (2 × 10^6^ cells/mL in HBSS) were incubated on poly-L-lysine-treated glass coverslips in a 24-well plate and treated or not with ArtinM or PMA, followed by incubation at 37°C for 6 h. Samples were collected, by gently removing coverslips, fixed with 3% paraformaldehyde (20 min at room temperature), and blocked in PBS supplemented with 3% FCS for 1 h at room temperature. Coverslips incubated overnight at 4°C with anti-neutrophil elastase antibody (ab21595; Abcam; 1:200) were washed and incubated for 1 h at room temperature with Alexa Fluor488 goat anti-rabbit IgG secondary antibody (Molecular Probes; 1:1000), and then washed and mounted with ProLong Antifade containing DAPI (Molecular Probes). Images were obtained using a Leica CTR 6000 fluorescence microscope, with Leica Application Suite software (Wetzlar, Germany).

### Assessment of neutrophil morphology

To assess cellular morphology, freshly isolated human neutrophils (2 × 10^6^ cells/mL in RPMI-1640) were treated with ArtinM, IL-8 (25 nM), Lysis Buffer (ammonium chloride 70 mM, Tris 10mM), or non-treated, and incubated during 3 and 20 h at 37°C in 5% CO_2_. After incubation, the cells were detached, as described above for MPO detection, cytocentrifuged onto a microscope slide using a Cytospin 3 cytocentrifuge (Thermo Shandon, USA), Diff-Quick-stained (Laborclin, Brazil) and examined using light microscopy (Olympus BX50—Olympus América INC, USA). Photomicrographs were taken with a Nikon DXM-1200 camera (Nikon instruments, USA).

### DNA fragmentation analysis

DNA electrophoresis was performed to determine the effects of ArtinM treatment on neutrophils DNA degradation. The analysis was performed using a kit, the Wizard SV genomic DNA purification system (Promega Corporation, USA). Briefly, after 24 and 48 h of incubation, 4 × 10^6^ cells were washed twice with PBS and lysed, and genomic DNA was isolated. The extracted DNA was quantified using the NanoVue Plus spectrophotometer (GE Healthcare, USA). A sample of 1 μg of DNA was analyzed using 1.5% agarose gel electrophoresis and stained with ethidium bromide. The DNA was then visualized under UV light on ChemDoc MP Imaging System and photographed using ImageLab software v.4.0 (both from Bio-Rad Laboratories, USA). Regarding the analysis of DNA fragmentation, the band area was quantified using ImageJ Software, and represented graphically in pixels^2^.

### Phosphatidylserine exposure assessment

Neutrophil apoptosis was assessed by Annexin V staining [33]. Human neutrophils (2 × 10^6^ cells/mL in RPMI-1640), were treated with ArtinM, IL-8, Lysis Buffer, or non-treated, and co-incubated or not with *L*. *major* during 3 and 20 h at 37°C in 5% CO_2_. After incubation, the cells were detached, as described above (MPO detection), washed once and suspended in 100 μL of annexin V binding buffer (140 mM NaCl, 2.5 mM CaCl_2_, 1.5 mM MgCl_2_, and 10 mM HEPES, pH 7.4), containing annexin V-^FITC or PE^ (1 μg/mL) for 15 min. Some assays were performed on neutrophils infected with green-fluorescent *L*. *major* forms (mβT3-LV39 strain), in order to detect if the dying cells are the ones infected. Immunofluorescence staining was analyzed by flow cytometry, using a Guava EasyCyte Mini instrument (Millipore, USA).

### Disruption of mitochondrial transmembrane potential

Human neutrophils (2 × 10^6^ cells/mL in RPMI-1640) were treated with ArtinM, IL-8, Lysis Buffer, or non-treated, and incubated during 3 and 20 h at 37°C in 5% CO_2_. JC-1 staining solution (10 μM) was then added to the wells and incubated at 37°C for 15 min. The fluorescent intensity for monomeric forms of JC-1 was measured using the FLx800 Fluorescence Microplate Reader (BioTek Instruments, USA; excitation 485 nm, emission 528 nm).

### Statistical analysis

Statistical analyses were performed by Student t test, one way ANOVA followed by Bonferroni's post-test, and two way ANOVA followed by Bonferroni's post-test (all using GraphPad Prism software version 6; GraphPad), as indicated at the graph. The p values <0.05 were deemed statistically significant.

## Results

### ArtinM enhances uptake and killing of *L*. *major* by neutrophils

To evaluate whether neutrophil activation by ArtinM contributes to its protective effect against intracellular pathogens [21], we compared the internalization and elimination of *L*. *major* by neutrophils that were pre-treated or were not pre-treated with ArtinM.

**Fig 1:**
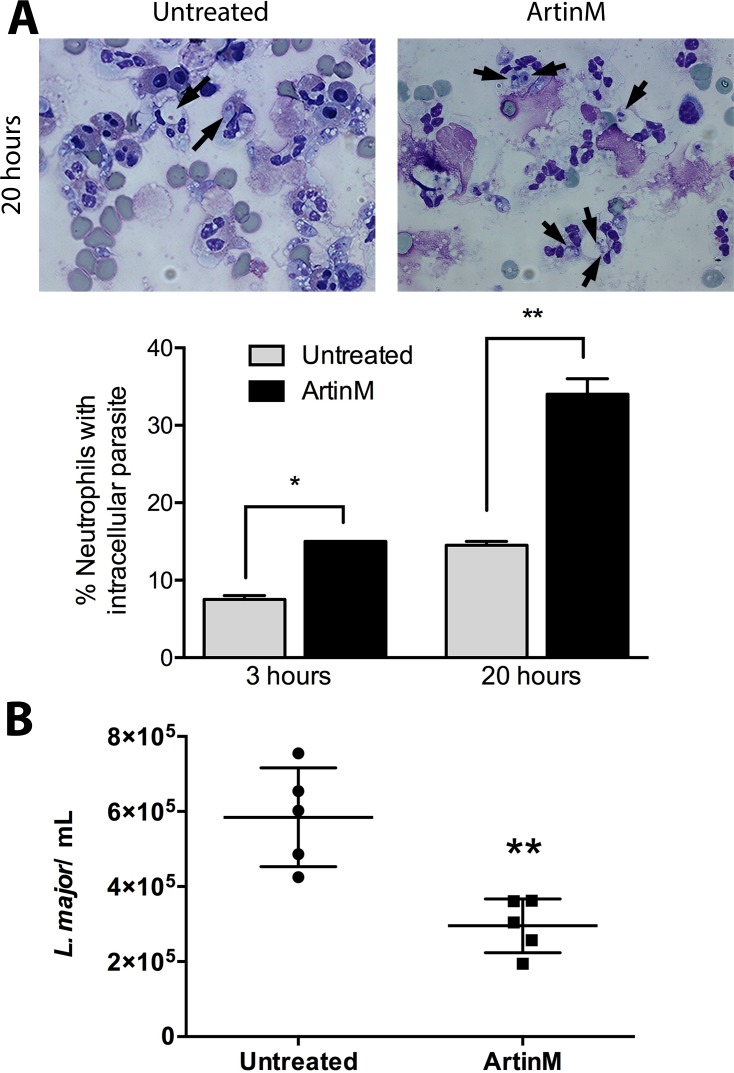
ArtinM enhances *L*. *major* uptake and killing by human neutrophils. Human neutrophils were treated or were not treated with ArtinM, and infected with *L*. *major* promastigotes (MOI 3:1). **A–*L*. *major* uptake.** After incubation for 3 and 20 h, the cells were centrifuged on slides and stained for evaluation by light microscopy. Arrows indicate internalized parasites. In the graphic, data are expressed as the mean of neutrophils with intracellular parasites ± SD. * p<0.05; ** p<0.01 in comparison to untreated cells at the same period. One way ANOVA followed by Bonferroni's post-test. **B–*L*. *major* killing.** After 3 h of infection the cultures were washed to discard uninternalized parasites, fed with Schneider`s medium and cultured for additional 48 h. Motile promastigotes were counted, and the data are expressed as mean of viable parasites ± SD. ** p<0.002: compared to untreated, Student *t* test. Each assay was carried out in triplicate. The shown data are representative from three different experiments.

We assessed the effect of ArtinM treatment on the frequency of neutrophils with internalized parasites. At 3 h after *in vitro* infection, the number (15±0%) of ArtinM-treated neutrophils with internalized parasites was 88% higher than the number (7.5±0.7%) verified in untreated neutrophils (Fig 1A). The difference increased 140% (14.5±0.7% *vs* 34±2.8%) when the same assay was performed 20 h after infection (Fig 1A).

We also evaluated the leishmanicidal activity of the ArtinM-treated neutrophils. Cells were washed at 3 h post-infection and cultured in Schneider`s medium for additional 48 h. At this point, the number of mobile parasites (2.9x10^5^±0.7x10^5^) recovered from the culture of ArtinM-treated neutrophils was, on average, 50% lower than the number of viable parasites (5.8x10^5^±1.3x10^5^) recovered from untreated neutrophils (Fig 1B).

In conclusion, our data show that ArtinM treatment increased the ability of neutrophils to eliminate *L*. *major*, a fact that favors the idea that neutrophils can contribute to the protective effect of ArtinM against infection.

### ArtinM alters the pattern of cytokine production by *L*. *major*-infected neutrophils

**Fig 2:**
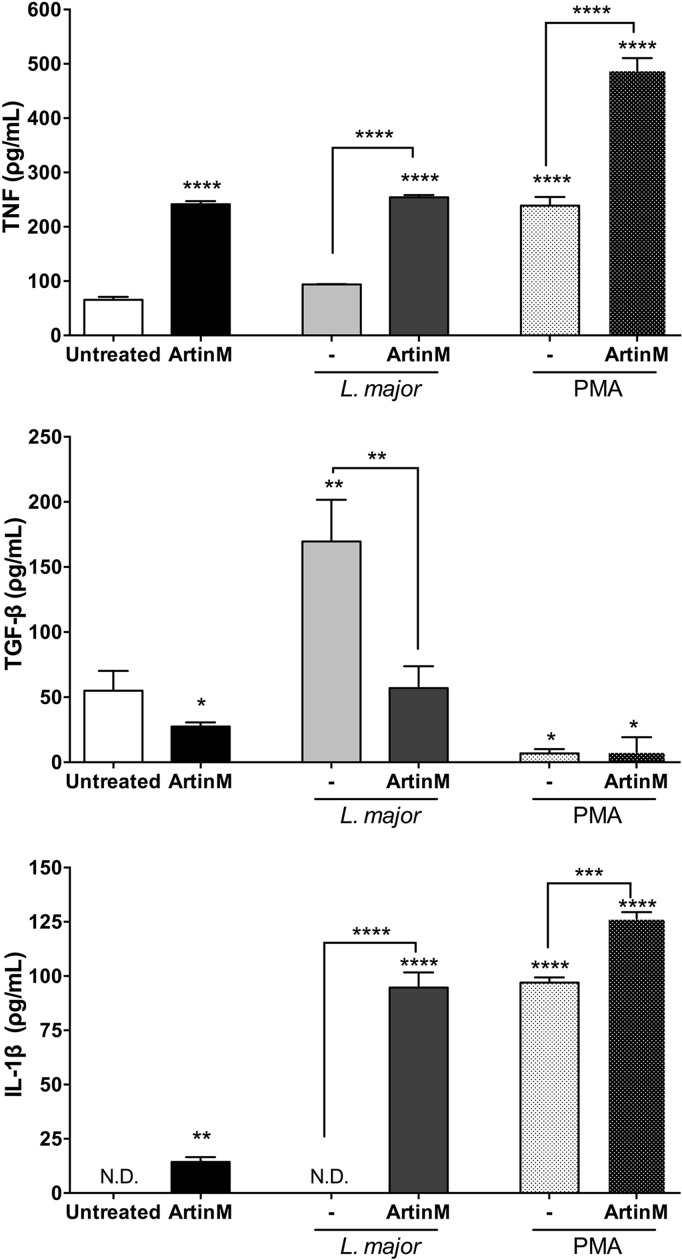
ArtinM stimulates the production of inflammatory cytokines by *L*. *major*-infected or uninfected neutrophils. Human neutrophils were treated with or without ArtinM or PMA, and either infected or not infected with *L*. *major* promastigotes (MOI 3:1). At 20 h post-infection, the supernatant of these cells was collected for cytokine measurement by ELISA. **A–TNF, B–TGFβ-1 and C–IL-1β** concentrations are expressed as mean ± SD. * p<0.05; **** p<0.001 in comparison to untreated cells, or as indicated. One way ANOVA followed by Bonferroni's post-test. ND stands for “not detected”. ELISA assays were carried out in triplicate. The shown data are representative from three different experiments.

The prominent production of inflammatory or anti-inflammatory cytokines by neutrophils contributes to an appropriate microenvironment for *L*. *major* elimination or survival, respectively [34,35]. We quantified the TNF, TGF-β, and IL-1β cytokines in the supernatant of non-treated or ArtinM-treated neutrophils that were infected or not with *L*. *major*. Twenty h after ArtinM-treatment, the supernatant contained 7-fold higher concentration of TNF (241.7±5.59 ρg/mL) than the supernatant of untreated neutrophils (65.65±5.47 ρg/mL). A similar level was detected when the assay was performed with *L*. *major* infected neutrophils (245.77±3.93 ρg/mL and 93.77±0.68 ρg/mL—Fig 2A). On the other hand, TGF-β production was 3-fold augmented in *L*. *major*-infected neutrophils (169.6±32.01 ρg/mL), while ArtinM-treated cells, infected (56.99±16.73 ρg/mL) or not (27.51±3.14 ρg/mL), released levels that were similar to the ones verified for untreated cells (54.94±15.27 ρg/mL—Fig 2B). IL-1β was not detected in the supernatant of untreated or *L*. *major* infected neutrophils. In contrast, we detected IL-1β production by ArtinM-treated neutrophils (14.37±2.19 ρg/mL), which increased by 6-fold when these ArtinM-treated cells were infected with *L*. *major* (94.67±7.01 ρg/mL—Fig 2C). Taken together, our results show that ArtinM treatment enhances the production of TNF and IL-1β, but not of TGF-β by human neutrophils. Instead of the high levels of TGF-β produced by *L*. *major*-infected neutrophils, the ArtinM pre-treated neutrophils released higher levels of TNF and IL-1β.

### ArtinM induces neutrophil degranulation

Releasing of the contents of neutrophil granules contributes to elimination of pathogens [36]. In the case of *L*. *major* infection, elastase released by azurophilic granules of neutrophils plays an outstanding protective role in host responses [37]. Here, we examined the release of neutrophil elastase (NE) and myeloperoxidase (MPO) by cells that were treated or not with ArtinM. The occurrence of neutrophil degranulation was verified by detection of (1) decreased intracellular content of MPO, using flow cytometry analysis, and (2) by augmented levels of NE in the cell supernatant, assessed by enzymatic activity quantification.

**Fig 3:**
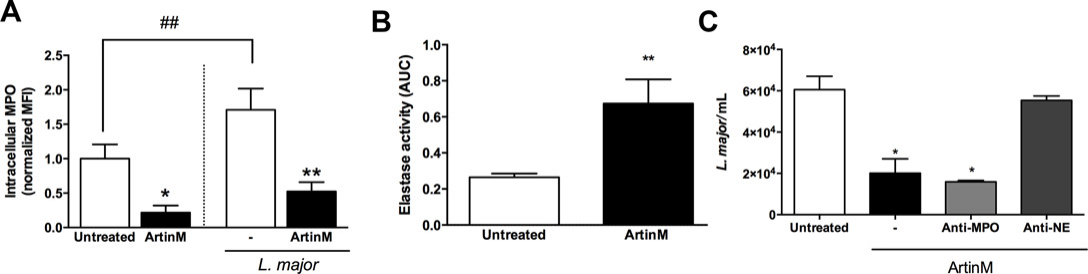
ArtinM stimulates the degranulation of *L*. *major*-infected or uninfected neutrophils. **A–Intracellular levels of myeloperoxidase.** Human neutrophils were incubated for 20 h with ArtinM or medium only (untreated), and infected or not infected with *L*. *major* promastigotes (MOI 3:1); cells were permeabilized and reacted with anti-MPO^PE^. Cells were analyzed by flow cytometry and data were expressed as normalized mean ± SD of the median fluorescence intensity. * p<0.02, **p<0,01. Student's *t* test. **B–Elastase releasing.** Human neutrophils were stimulated with ArtinM or medium only (untreated); Cell supernatants were monitored for 30 min for enzymatic activity, using the substrate N-succinyl-Ala-Ala-Val-p-nitroanilide. The NE activity was expressed as the area under curve (AUC) ± SD. ** p<0.05 in comparison to untreated cells. Student's *t* test. **C–Elastase leishmanicidal activity.** Supernatants from neutrophils treated for 1 h or not treated with ArtinM were incubated for 30 min (or not incubated) with anti-MPO or anti-NE antibodies (both 1:500). Next, supernatants were added to *L*. *major* culture (1x10^5^), in Schneider’s medium. After 24 h, live parasites were quantified by reaction with Alamar Blue. Data are expressed as the number of viable parasites ± SD. * p<0.05 in comparison to the supernatant from untreated cells. One way ANOVA, followed by Bonferroni's post-test. Each assay was carried out in duplicate. The shown data are representative from three different experiments.

The intracellular MPO content in ArtinM-treated neutrophils was 4-fold lower than that verified in untreated cells (Fig 3A). Consistent with this, 2-fold higher levels of NE activity were detected in the supernatant of ArtinM-treated neutrophils in comparison to that observed in the supernatant of untreated cells (Fig 3B). This last assay was actually performed to verify the period necessary for occurrence of neutrophil degranulation in response to ArtinM, and the highest NE activity was detected shortly (5–60 min) following treatment, even when the concentrations used were as low as 312 ng/mL of lectin, as demonstrated by a time-course assay (S2 Fig).

We also examined the neutrophil degranulation after *L*. *major* infection. Regarding MPO intracellular content, we verified that the infection *per se* was able to inhibit the degranulation of untreated cells (70%—Fig 3A). In contrast, the intracellular levels of MPO decreased drastically in the ArtinM-treated neutrophils (3-fold), showing that the lectin promotes degranulation even in *L*. *major* infected neutrophils (Fig 3A). Therefore, ArtinM induces degranulation of both uninfected and infected neutrophils.

### Elastase contributes to the augmented leishmanicidal activity induced by ArtinM on neutrophils

To assess the leishmanicidal activity of the neutrophil secreted products, we incubated parasites with the supernatant of cells that were pre-stimulated with ArtinM. The number of viable *L*. *major* was 66% lower following incubation with the supernatant of ArtinM-treated neutrophils (2.0x10^4^±0.6x10^4^) than with the supernatant of untreated cells (6.0x10^4^±6.4x10^4^). In order to evaluate the specific contribution of MPO and NE, released by azurophilic granules, to the observed leishmanicidal activity, the supernatant of ArtinM-treated neutrophils was pre-incubated with antibodies specific to MPO or NE, and then added to the parasite suspensions. Our results show that anti-MPO had no effect on the number of viable *L*. *major* (1.5x10^4^±0.07x10^4^), whereas anti-NE antibodies inhibited the leishmanicidal activity provided by the supernatant of ArtinM-treated neutrophils once the number of parasites recover in this condition (5.5x10^4^±0.2x10^4^) was similar to that found on the supernatant coming from untreated neutrophils (Fig 3C). These data suggest that the leishmanicidal activity of ArtinM-treated neutrophils is due, at least partially, to an NE-mediated mechanism.

### ArtinM inhibits the formation of Neutrophil Extracellular Traps induced by *Leishmania major*

**Fig 4:**
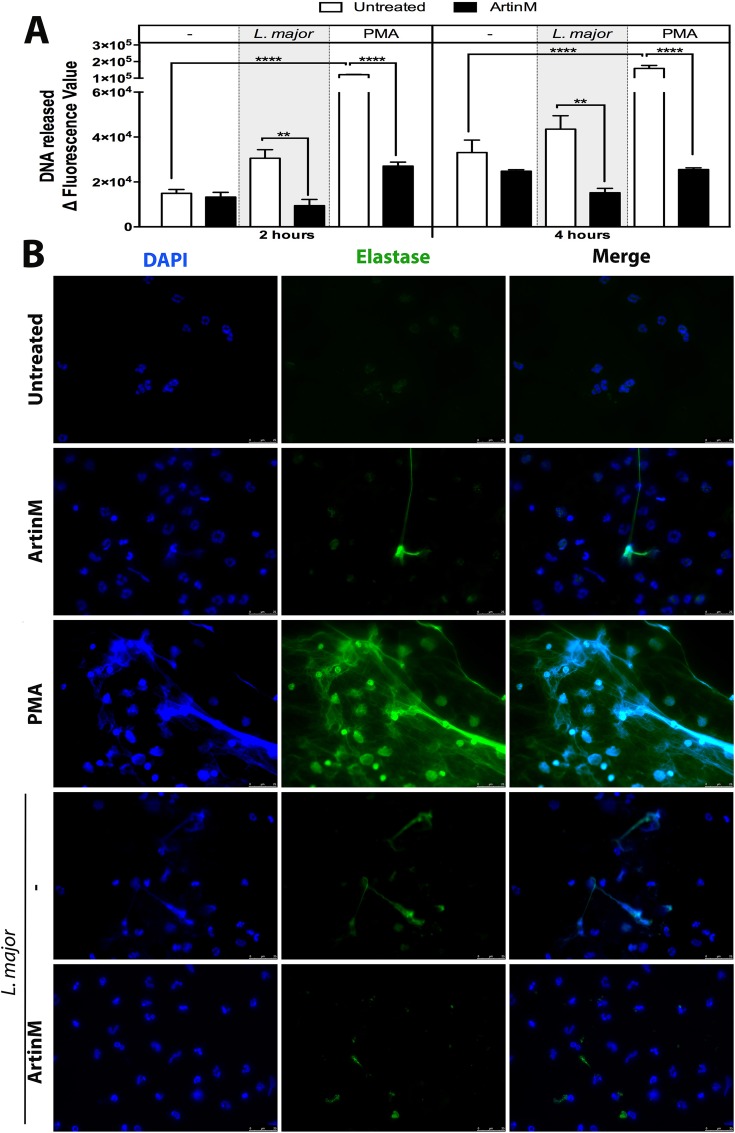
ArtinM inhibits NET formation induced by *L*. *major*. Human neutrophils were incubated with ArtinM, PMA, ArtinM+PMA, or medium (untreated), and were either infected or not infected with *L*. *major* promastigotes (3:1). **A–DNA quantitation.** DNA released at 2 and 4 h after treatment/infection was quantified in the cell supernatants by fluorescence detection (Ex./Em. 480/520 nm) after reaction with SYTOX green. Data are expressed as a mean of fluorescence intensity ± SD. **p<0.01; ***p<0.001; **** p<0.0001. Two way ANOVA followed by Bonferroni's post-test. DNA quantitation assays were performed in triplicate and, the data shown are representative of four independent experiments. **B–NET immunofluorescence detection.** Neutrophils were plated onto poly-l-lysine coated slides, treated or untreated with PMA or ArtinM, and either infected or not infected with *L*. *major* promastigotes (MOI 3:1). After 6 h, the cells were stained with DAPI (DNA blue stain) and with anti-NE antibody (green). Merged images confirmed NETs by colocalization of staining. NET immunofluorescence detection assays were performed in duplicate and the data shown are representative of three independent experiments.

Besides killing microbes through its direct enzymatic activity [38–40], NE also contributes to the formation of neutrophil extracellular traps (NET) [41], which constitutes a mechanism for capture and kill microorganisms [42] and for host tissue damage [43]. To investigate whether neutrophil treatment with ArtinM could result in NET formation, the DNA concentration was measured in the supernatant of neutrophils, 2 and 4 h after ArtinM-treatment. The DNA levels detected in the supernatant of ArtinM-treated cells were as low as those found in untreated neutrophils. In contrast, neutrophil stimulation with PMA (positive control) resulted in the detection of high DNA levels (> 5- fold, Fig 4A), at both periods analyzed. When we assayed *L*. *major*-infected neutrophils, we observed that untreated cells formed NET, in concordance with previous reports [44], but, in contrast, ArtinM-treated cells do not form NET (Fig 4A). The microscopic observation of the assayed cells was consistent with the DNA measurement in the neutrophil supernatant (Fig 4B). Therefore, we concluded that the ArtinM treatment does not induce NET formation and inhibits the formation of NET that follows *L*. *major* infection.

### ArtinM induces ROS production only by infected neutrophils

Once we had verified that ArtinM triggers intense neutrophil activation not associated with NET formation, we evaluated whether lectin induces the production of ROS, which is directly implicated in NET formation [45]. We monitored the levels of ROS following exposure to the ArtinM stimulus, through reactions with luminol or lucigenin, which are oxygenated by H_2_O_2_ (and its derived species), or by superoxide anion, respectively [46].

**Fig 5:**
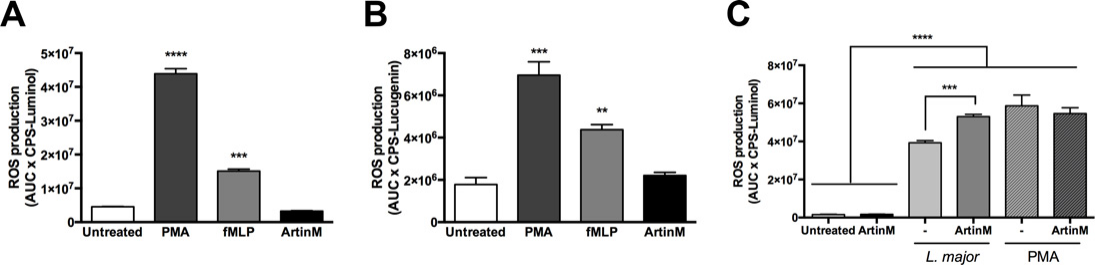
ArtinM augments ROS production by *L*. *major*-infected neutrophils. Human neutrophils were treated with PMA, fMLP, ArtinM or medium (untreated). ROS production was quantified by reaction with luminol (**A**) and lucigenin (**B**), producing chemiluminescent photons (CPS). Data on PMA stimulation or *L*. *major* promastigotes infected neutrophils (3:1), which were treated or not treated with ArtinM, are shown in panel **C**. The amounts of ROS released are expressed as mean AUCxCPS ± SD. ** p<0.01; *** p<0.001; **** p<0.0001. One way ANOVA followed by Bonferroni's post-test. Each assay was carried out in triplicate. The data shown are representative of three independent experiments.

As shown in Fig 5, ArtinM-treated neutrophils, as well as untreated control cells, did not produce ROS since low and stable levels were demonstrated by both luminol- and lucigenin-chemiluminescence detection. In contrast, neutrophils stimulated with PMA or fMLP (positive controls) triggered high ROS production (Fig 5A and 5B).

Although ArtinM did not induce ROS production, it did not inhibit the production induced by other agents, a fact that was demonstrated by the observation that following the incubation with ArtinM, neutrophils responded to PMA- or fMLP-stimulus, with ROS levels similar to the ones measured in cells that were not pre-incubated with ArtinM (Fig 5C and S3 Fig).

The luminol-monitored ROS detection revealed that a higher amount (26-fold augmented) was produced by infected (3.93x10^7^±1.0x10^6^ AUCxCPS) compared to uninfected neutrophils (1.49x10^6^±0.2x10^6^ AUCxCPS). When the infected cells were pre-treated with ArtinM, the ROS production increased by 25% (5.3x10^7^±1.1x10^6^ AUCxCPS—Fig 5C). Taken together, in spite of not inducing uninfected cells to produce ROS, ArtinM enhances ROS production by *L*. *major* infected neutrophils, providing a mechanism that rapidly eliminates the parasite.

### ArtinM treatment prolongs neutrophil survival

The sustained neutrophil activation induced by ArtinM, which was detected even 20 h after treatment (S4 Fig), as well as the absence of NET formation and ROS production, motivated us to evaluate the survival rate of ArtinM-treated neutrophils. Several methods were used to assess the occurrence of cell death, such as the analysis of neutrophil morphological changes, fragmentation of genomic DNA, phosphatidylserine (PS) exposure, and disruption of the mitochondrial transmembrane potential.

**Fig 6:**
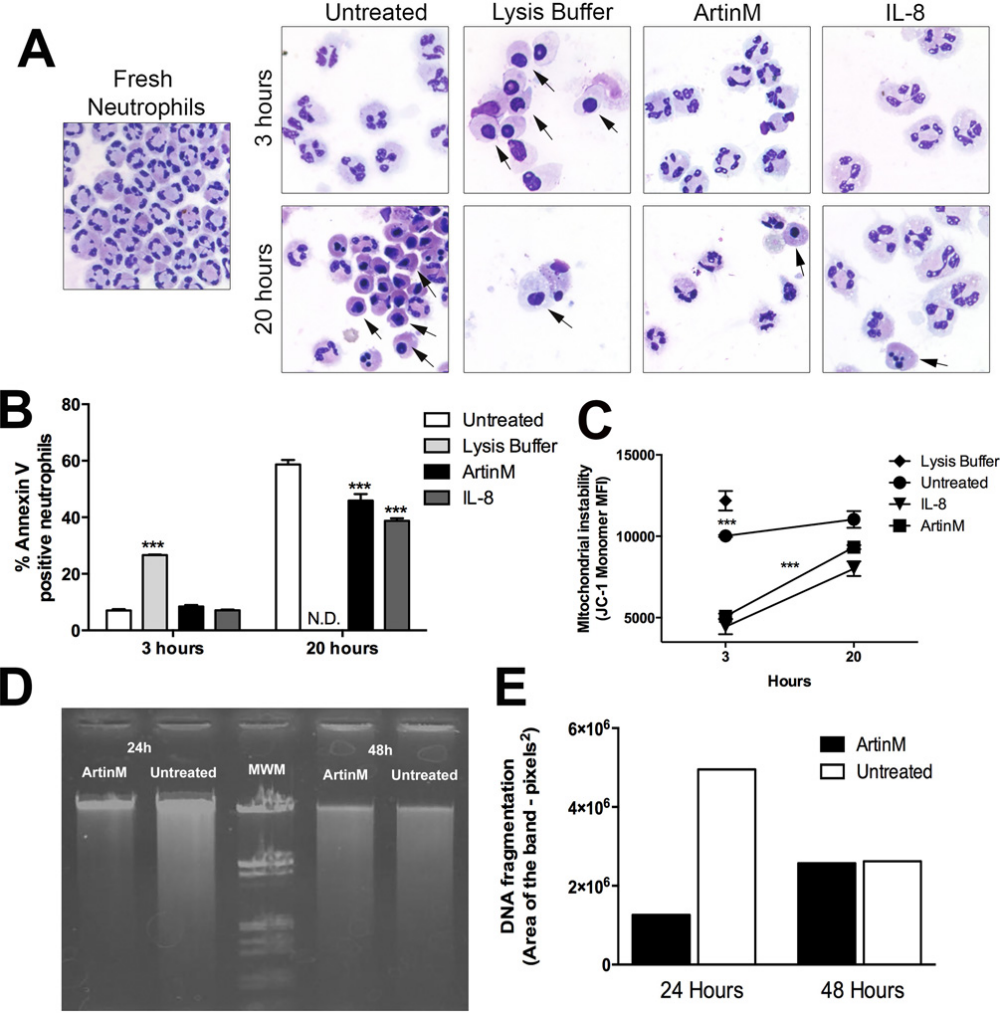
ArtinM treatment postpones apoptosis of uninfected neutrophils. Human neutrophils were incubated (indicated period) with medium (untreated), lysis buffer, ArtinM or IL-8. **A–Neutrophil morphology.** Neutrophils were cytocentrifuged and stained for evaluation by light microscopy. Arrows indicate neutrophils with nuclear condensation. **B–Phosphatidylserine exposure.** Neutrophils were labeled with Annexin V^-FITC^ and analyzed by flow cytometry. Data are expressed as mean of percentage of AnnexinV^+^ neutrophils ± SD. *** p<0.001 in comparison with the untreated cells, two way ANOVA followed by Bonferroni's post-test. ND stands for “not detected”. **C**–**JC-1 monomer detection.** Neutrophils were incubated with JC-1 probe and green fluorescence detection (Ex/Em = 485/528) was performed at 3 and 20 h after treatment. Data are expressed as mean of fluorescence intensity ± SD. *** p<0.01 comparing with untreated curve, two way ANOVA followed by Bonferroni's post-test. **D and E—Electrophoretic detection of DNA fragmentation.** Neutrophils were incubated for 24 and 48 h with ArtinM or medium (untreated). Their genomic DNA was analyzed by gel electrophoresis. Images are shown on panel **D.** Panel **E** represents the band area in pixels^2^, regarding the electrophoretic detection of DNA fragmentation (ImageJ Software). Each assay was carried out in triplicate. The shown data are representative from three different experiments.

The morphology of neutrophils was evaluated by optical microscopy. At 3h post-treatment, the microscopic features of ArtinM- or IL-8-treated cells, as well as of untreated cells, were typical of live neutrophils. At the same time point, cells treated with lysis buffer, as expected, showed remarkable nuclear condensation, as usually observed in apoptotic neutrophils [5, 6]. After 20 h, nuclear condensation was verified in untreated cells, while the ArtinM- or IL-8-treated ones preserved their original morphology (Fig 6A).

Flow cytometry analysis of neutrophils that were incubated for 3 h with ArtinM (8.43±1.0%), or IL-8 (7.13±0.3%) showed levels of PS exposure that were similar to the ones detected in untreated cells (6.98±0.8%) and were significantly lower (4-fold) than those detected in lysis buffer-treated cells (26.64±0.3%—Fig 6B). After 20 h, high exposure of PS was detected on untreated cells (58.66±2.7%) whereas 20% and 34% lower levels were measured in ArtinM (45.89±3.9%) and IL-8 (38.8±1.4%) -treated cells, respectively. The analysis of neutrophils 20 h after treatment with lysis buffer was barred due to the scarce number of remaining cells (Fig 6B).

ROS production, mostly mitochondrial, is implicated in the initiation of cell apoptosis [47]. Since disruption of the mitochondrial trans-membrane potential (ΔΨ) is one of the earliest intracellular events occurring in apoptotic cells, we examined mitochondrial instability through the detection of JC-1 monomer. Fig 6C shows that the treatment of neutrophils with ArtinM (5091±276.5 MFI) or IL-8 (4446±800.8 MFI) favored mitochondrial stability, since at 3 h post-treatment, the levels of JC1 monomer were at least half of those detected in untreated (10020±105.4 MFI) or lysis buffer-treated neutrophils (12186±1037 MFI). At 20 h, although JC1 monomer levels increased in ArtinM (9331±211.2 MFI) or IL-8 (8066±635 MFI) treated neutrophils, they remained significantly lower (-15%) than those detected in untreated neutrophils (11035±891 MFI). JC1 monomers were not detected in the rare neutrophils remaining 20 h after treatment with lysis buffer.

The electrophoresis analysis of DNA showed that ArtinM-treated neutrophils displayed at least two-fold less fragmented DNA than untreated cells at 24 h of incubation. At 48 h of incubation, treated and untreated neutrophils displayed similar DNA fragmentation. (Fig 6D and 6E).

To summarize, these data demonstrate that ArtinM treatment of human neutrophils postponed apoptosis, as shown by delayed disruption of the mitochondrial trans-membrane potential, DNA fragmentation, nuclear condensation and PS exposure. Altogether, these results revealed that ArtinM treatment prolongs neutrophil survival.

### ArtinM triggers early apoptosis of *L*. *major* infected neutrophils

**Fig 7:**
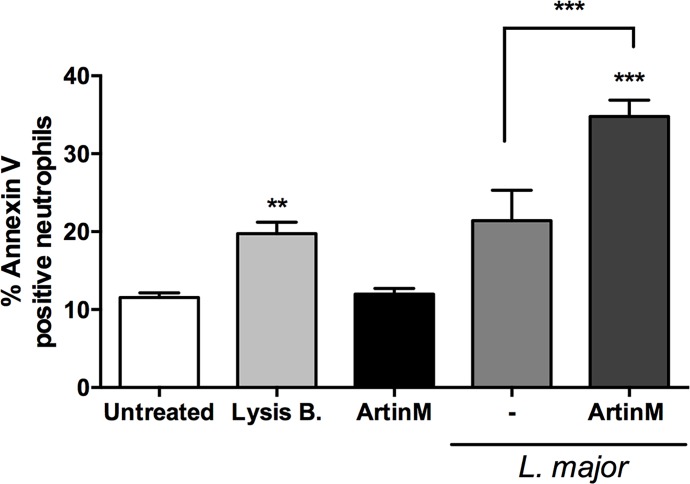
ArtinM induces early apoptosis of *L*. *major* infected neutrophils. Human neutrophils were incubated with medium (untreated), lysis buffer, ArtinM, or IL-8, and infected or not with *L*. *major* promastigotes (MOI 3:1). At 3 h post infection, cells were labeled with Annexin V^-FITC^ and analyzed by flow cytometry. Data are expressed as mean of percentage of AnnexinV^+^ neutrophils ± SD. ** p<0.01; *** p<0.001 in comparison to untreated cells, or as indicated. Two way ANOVA followed by Bonferroni's post-test. Each assay was carried out in triplicate. The shown data are representative from three different experiments.

We found that ArtinM prolongs neutrophil survival, a fact that is considered to facilitate *L*. *major* infection [48]. Then, we analyzed the PS exposure on ArtinM-treated neutrophils that were infected with *L*. *major*. The ArtinM effect, exerted 3 hours after stimulation on the neutrophils PS exposure, at least doubled when the cells were infected with *L*. *major*, compared to uninfected cells (34.8±2.07% *vs* 11.97±0.74%, Fig 7). In addition, these infected/ArtinM-treated neutrophils exhibited 40% higher PS exposure than the untreated/infected neutrophils (34.8±2.07% *vs* 21.41±3.92%, Fig 7). Uninfected cells, treated or not with ArtinM, were used as control, at the same time point. The obtained results (11.97±0.74% and 11.55±0.61%) were close to those shown in Fig 6B (3h). Therefore, ArtinM stimulus delays the death of non-infected neutrophils (at 20 hours’ time point, Fig 6B), and accelerates the death of *L*. *major*-infected neutrophils (Fig 7), favoring the parasite elimination.

Some assays performed by infecting neutrophils with fluorescent *L*. *major* forms allowed verifying that 91% of the AnnexinV labeled cells were infected. A close proportion (89%) was verified among ArtinM-treated cells as well (S5 Fig). We therefore concluded that apoptotic neutrophils were most frequently *L*. *major* infected cells, and that ArtinM did not change this distribution.

## Discussion

The immunomodulatory lectin ArtinM induces Th1 immune response and confers resistance to intracellular pathogens [21], without causing apparent tissue damage. The response to ArtinM is induced by its interaction with glycoconjugates on several immune cells, namely, macrophages, dendritic cells, neutrophils, mast cells and lymphocytes [10,11,13,16,17,24–26,49]. Considering that neutrophils constitute a two-edged sword, accounting for resistance to pathogens and also for tissue injury, a full exploration of the neutrophil responses is required to envisage a possible application of ArtinM, or its analogues, as immunomodulatory agent. In this study, we verified that ArtinM enhances the neutrophils ability of eliminating the intracellular pathogen *L*. *major*. The parasite killing was associated with strong neutrophil activation, not accompanied by NET formation, which is known to cause tissue damage.

Although macrophages are the definitive refuge for *Leishmania* species in the host, neutrophils are considered by many as transitional shelters for the few invader parasites that survive the toxic extracellular milieu [50]. This idea is based on the observation that in the early stages of *L*. *major* infection, caused by the bite of an infected sandfly, large amounts of neutrophils are attracted to the site. Since a low number of macrophages reside in the invaded tissue and the recruited neutrophils failed to kill *L*. *major*, the authors concluded that the invading parasites depended on the rapidly recruited neutrophils to survive [51]. Once inside the neutrophils, *L*. *major* promastigotes postpone neutrophil apoptosis until 2 days [48] and are silently transferred to macrophages, without activating the immune response [51]. In opposition to this “Trojan horse” mechanism of parasite evasion, ArtinM accelerates the death of *L*. *major-*infected neutrophils, favoring the process of parasite elimination.

The production of the anti-inflammatory cytokine TGF-β favors the silent uptake of apoptotic cells by macrophages, whereas the production of pro-inflammatory mediators like TNF and IL-1β decreases after the uptake of apoptotic cells [52]. Consequently, the prominence of TGF-β correlates with permissibility to *L*. *major* infection, whereas high production of TNF is associated with resistance to infection [35]. Our observation that *L*. *major*-infected neutrophils largely augmented TGF-β production and diminished the secretion of pro-inflammatory mediators is consistent with previous demonstrations that neutrophils are important contributors toward providing the required microenvironment for parasite survival [34,53,54]. Notably, the treatment of neutrophils with ArtinM inverted this pattern by significantly diminishing TGF-β production and augmenting the secretion of pro-inflammatory cytokines. We found that ArtinM promoted IL-1β secretion by human neutrophils, which was maximum when the lectin was added to *L*. *major* infected neutrophils. This is a relevant finding because IL-1β maturation, as recently demonstrated, results from activation of the NLRP3 inflammasome, which is an innate platform that crucially restricts parasite replication. The NLRP3 inflammasome triggers inducible nitric oxide synthase (NSO2)-mediated production of NO, a potent leishmanicidal factor [55]. In addition, NLRP3 inflammasome activation is associated with ROS production [56], which is a major factor for *Leishmania* killing by human cells [57]. Indeed, macrophages derived from human monocytes do not produce NO after classical activation [58] or upon infection with *Leishmania* [59]. Thus, neutrophils, monocytes, and macrophages can control the parasites via ROS that are produced during the respiratory burst process [60]. By promoting ROS production and activating the NLRP3 inflammasome, ArtinM facilitates *Leishmania* killing, as shown in this work. Considering that the importance of IL-1β, derived from inflammasome, for conferring resistance to Leishmania infection was demonstrated for other species than *L*. *major* [55], we plan performing further experiments with *L*. *braziliensis*-infected neutrophils, to better assess the IL-1β relevance for the ArtinM-induced protection against the parasite.

Another trump against *Leishmania* infection provided by ArtinM treatment was the increased degranulation of human neutrophils. The intracellular levels of MPO were diminished in ArtinM-treated neutrophils, whereas the NE activity was augmented extracellularly. Both MPO and NE are stored in azurophilic granules, and their enzymatic activities are known to be implicated in the degradation of microorganisms in phagolysosomes. MPO is a key component of the oxidative burst, producing hypochlorous acid and other reactive oxidants [61], whereas NE is a serine protease that degrades the outer membrane of microorganisms, inducing their elimination [39]. NE and MPO have low capacity to be exocytosed from the neutrophils azurophilic granules during infectious and inflammatory processes [36,62–64]. We found that treatment with ArtinM increased the exocytosis of these granules, even when the cells were infected with *L*. *major*. Many authors attribute an important role to neutrophil degranulation in mounting a response that culminates in pathogen elimination or control [36,65,66]. Regarding NE, recent data revealed that its release into the extracellular environment induces macrophage activation via TLR4, which culminates in augmented internalization and elimination of *L*. *major* [67]. In the current work, we did not investigate the signaling through TLR4, nor the relationship with macrophages, but we demonstrated that the ArtinM-induced NE release was followed by elimination of *L*. *major* by human neutrophils (Figs 1 and 2). The released NE accounts for the ArtinM leishmanicidal effect, once the NE presence in the extracellular space correlates with *L*. *major* elimination, as indicated by experiments using anti-NE neutralizing antibodies. Although NE is a serine protease that promotes microbe killing, our study apparently constitutes the first demonstration of its leishmanicidal activity, a finding that surely deserves further investigation.

ArtinM-treated neutrophils, in sterile conditions, did not produce ROS, while other neutrophil activators such as fMLP and PMA induced rapid and intense responses. The *L*. *major* infection of neutrophils increased ROS production, which was even higher in ArtinM-treated neutrophils. It is well established that ROS provides an important mechanism to combat *L*. *major* [68], but is also able to cause tissue injury. The ROS production induced by ArtinM was restricted to infected cells whose life span was shortened by ArtinM treatment, allowing us to postulate that the ability of ROS to cause tissue damage could be reduced. The effect of ROS on parasite elimination could be preserved, since it is effective in the early stages of infection [69].

ROS production accounts for NET formation, as demonstrated by the observation that neutrophils treated with NADPH oxidase, a pharmacological inhibitor of ROS production, do not form NET in response to conventional stimuli [70]. We showed that ArtinM-stimulated neutrophils, either uninfected or infected with *L*. *major*, did not form NET. This means that ArtinM inhibits the NET formation induced by the infection itself [44] and additionaly by PMA The inhibition occurred in spite of NE release and ROS production, which favor NET formation and were verified to occur in ArtinM-treated neutrophils. NET formation constitutes a mechanism whereby neutrophils eliminate pathogens, including *L*. *amazonensis* promastigotes [71]. Other *Leishmania* spp, namely, *L*. *donovani*, *L*. *major*, *L*. *infantum*, and *L*. *mexicana*, escape from their toxicity despite NET formation and trapping by these web-like structures [44,72,73]. The fact that ArtinM, although inhibiting NET formation, facilitates *L*. *major* clearance by human neutrophils reinforces the idea that NET formation is not required for *L*. *major* elimination. On the other hand, the absence of NETs can favor host tissue integrity, since NET-associated proteases and granular proteins have been shown to damage host tissue [74].

**Fig 8:**
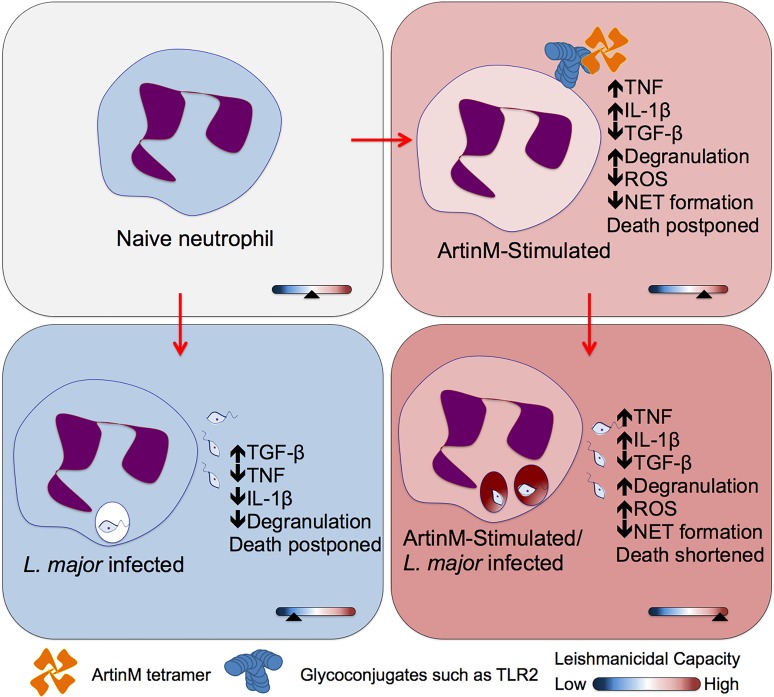
ArtinM increases the leishmanicidal capacity of human neutrophils: a model. Once infected with *L*. *major*, non-stimulated neutrophils produce high levels of TGF-β, and low levels of TNF, and IL-1β, which are associated with decreased cell degranulation and postponed cell death. In contrast, ArtinM-treated neutrophils become activated, produce high levels of TNF, and IL-1β, while TGF-b is reduced, and enhance degranulation. Collectively, these ArtinM induced responses augments the leishmanicidal capacity of neutrophils.

We conclude that ArtinM treatment of human neutrophils enhances the clearance of the intracellular pathogen *L*. *major*, through mechanisms that include: (1) production of inflammatory cytokines, i.e., high TNF and IL-1β, and virtual absence of TGF-β; (2) increased neutrophil degranulation; the released elastase promotes *L*. *major* elimination; (3) increased ROS production by infected neutrophils (4) shortened neutrophil survival. On the other hand, the host tissue integrity is favored by (1) the short period of ROS production and (2) the absence of NET formation. Fig 8 delineates our model of ArtinM effects on neutrophils during *L*. *major* infection, based on our results and assumptions.

## Supporting Information

S1 Fig Neutrophil purity analysis. Layered polymorphonuclear cells were analyzed for neutrophil purity. **A—**Cells were labeled with anti-CD16b^PE^ or isotype control^PE^ antibodies and analyzed by flow cytometry. **B–**Cells were cytocentrifuged and stained for morphology analyzes on light microscopy.
(TIF)

S2 Fig Time-course assay for detection of NE activity in the cell supernatants. Human neutrophils were treated with ArtinM (312,5–2.500 μg/mL), fMLP or medium (untreated). Cell supernatants were monitored for 30 min for enzymatic activity by using the substrate N-succinyl-Ala-Ala-Val-p-nitroanilide.
(TIF)

S3 Fig ArtinM does not block ROS production. Human neutrophils were pre-treated with ArtinM for 30 min and then treated with PMA or fMLP. ROS production was quantified by reaction with Luminol producing chemiluminescent photons (CPS). The kinetics of ROS production is shown. No significant differences were detected between pre-treated or not treated with ArtinM.
(TIF)

S4 Fig ArtinM promotes neutrophil polarization. Human neutrophils were 3 and 20 h incubated with ArtinM, IL8, or medium (untreated). **A—**Images from the wells of the plates were obtained by inverted light microscopy coupled with an image capturing system. **B–**The percentage of polarized cells was determined by using the ImageJ software. Data are expressed as mean of percentage of polarized cells analyzed in 3 different fields ± SD. * p<0.05: **p<0,01: *** p<0.001 in comparison to untreated cells at the respective time. Two way ANOVA, followed by Bonferroni's post-test.
(TIF)

S5 Fig AnnexinV^Phycoerythrin^ labelling of a human neutrophils culture incubated with green-fluorescent forms of *L*. *major* promastigotes (MOI 3:1). Flow cytometry analysis showed that about 90% of cells were double labelled, regardless the ArtinM treatment.
(TIF)
