# Supplementary figures and images for: Neutrophils Contribute to the Protection Conferred by ArtinM against Intracellular Pathogens: A Study on Leishmania major
Source: PLoS Negl Trop Dis. 2016 Apr 8;10(4):e0004609. doi: 10.1371/journal.pntd.0004609 (PMC4825989; doi:10.1371/journal.pntd.0004609)

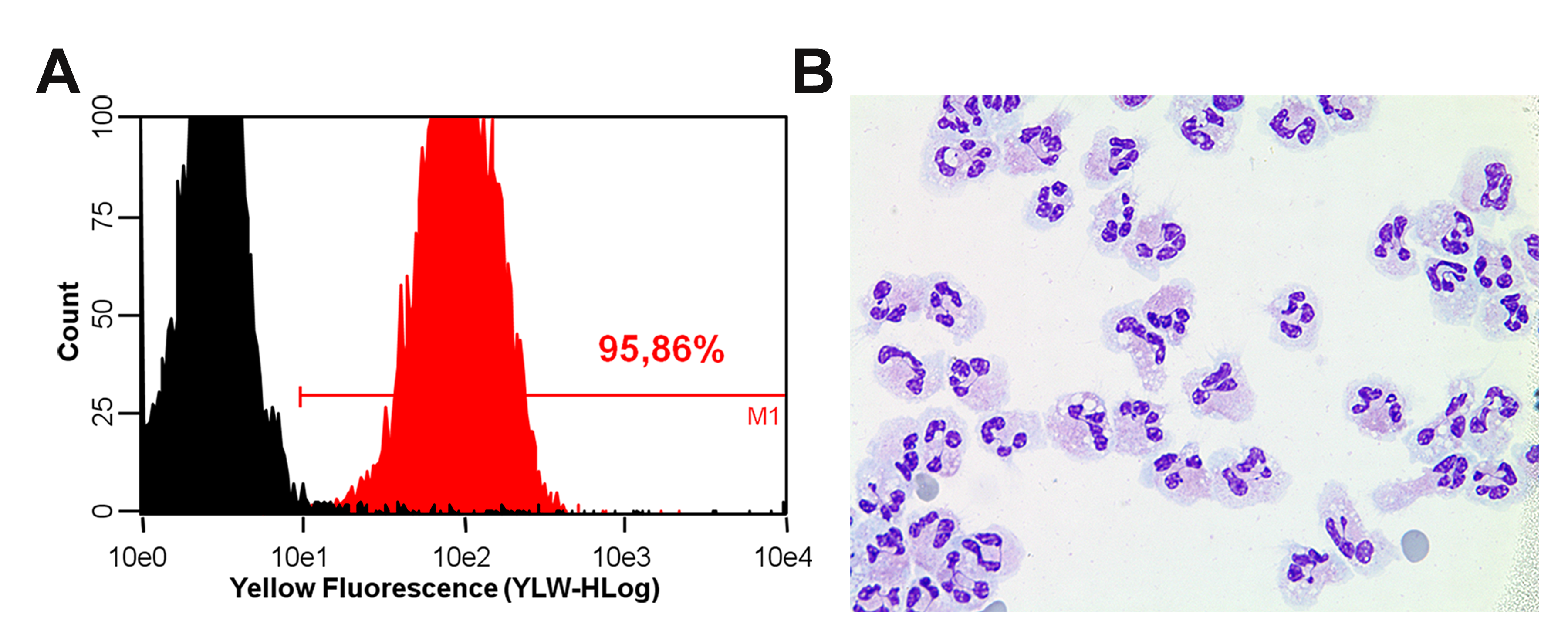

Supplement: S1 Fig — Layered polymorphonuclear cells were analyzed for neutrophil purity. A—Cells were labeled with anti-CD16bPE or isotype controlPE antibodies and analyzed by flow cytometry. B–Cells were cytocentrifuged and stained for morphology analyzes on light microscopy. (TIF) [file pntd.0004609.s001.tif]

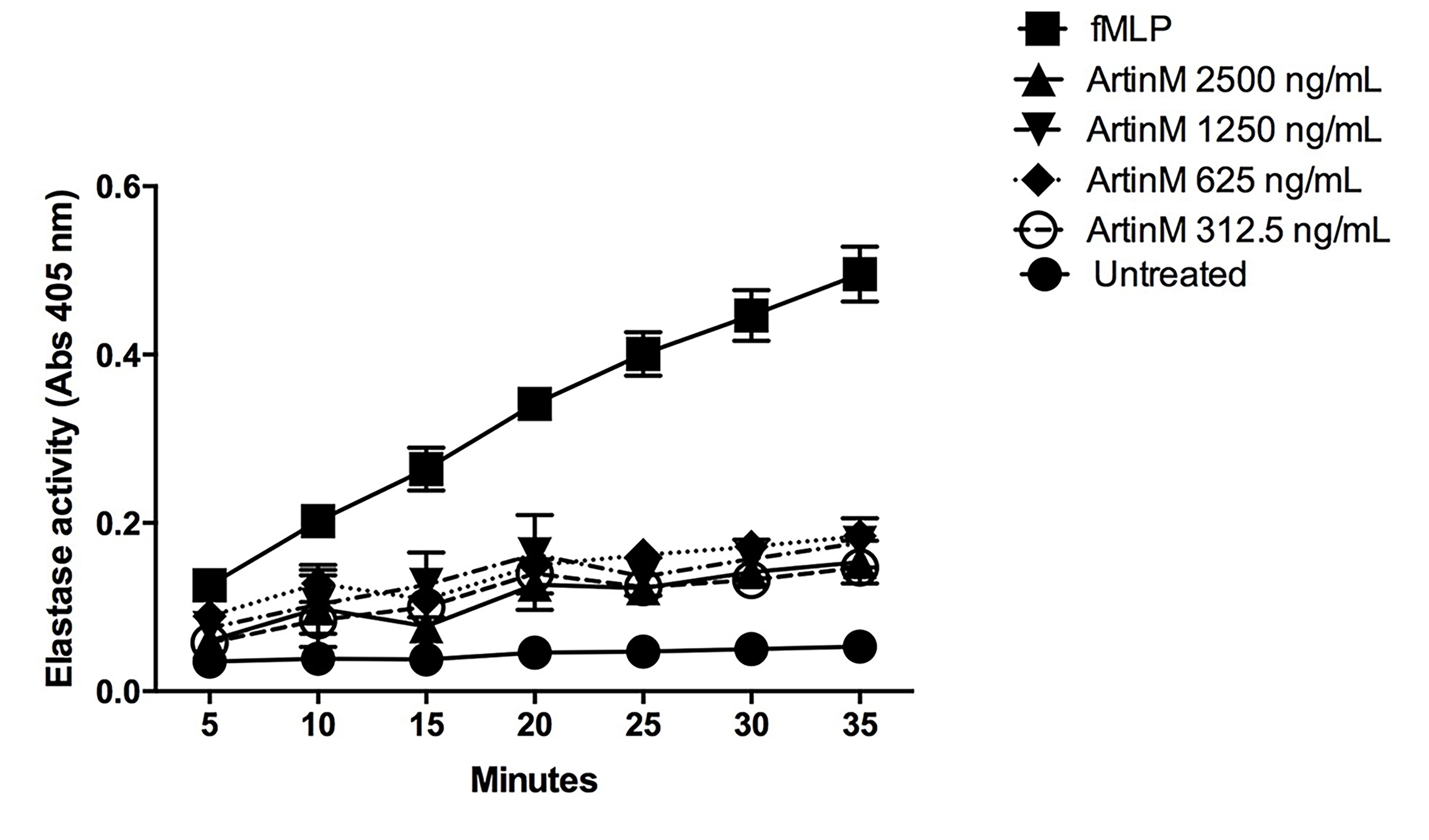

Supplement: S2 Fig — Human neutrophils were treated with ArtinM (312,5–2.500 μg/mL), fMLP or medium (untreated). Cell supernatants were monitored for 30 min for enzymatic activity by using the substrate N-succinyl-Ala-Ala-Val-p-nitroanilide. (TIF) [file pntd.0004609.s002.tif]

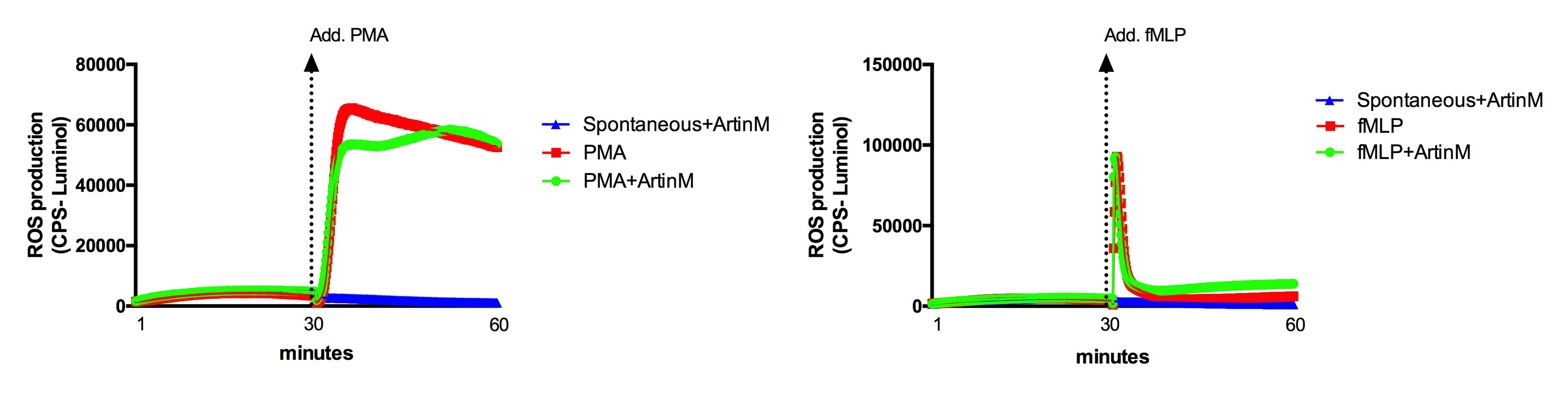

Supplement: S3 Fig — Human neutrophils were pre-treated with ArtinM for 30 min and then treated with PMA or fMLP. ROS production was quantified by reaction with Luminol producing chemiluminescent photons (CPS). The kinetics of ROS production is shown. No significant differences were detected between pre-treated or not treated with ArtinM. (TIF) [file pntd.0004609.s003.tif]

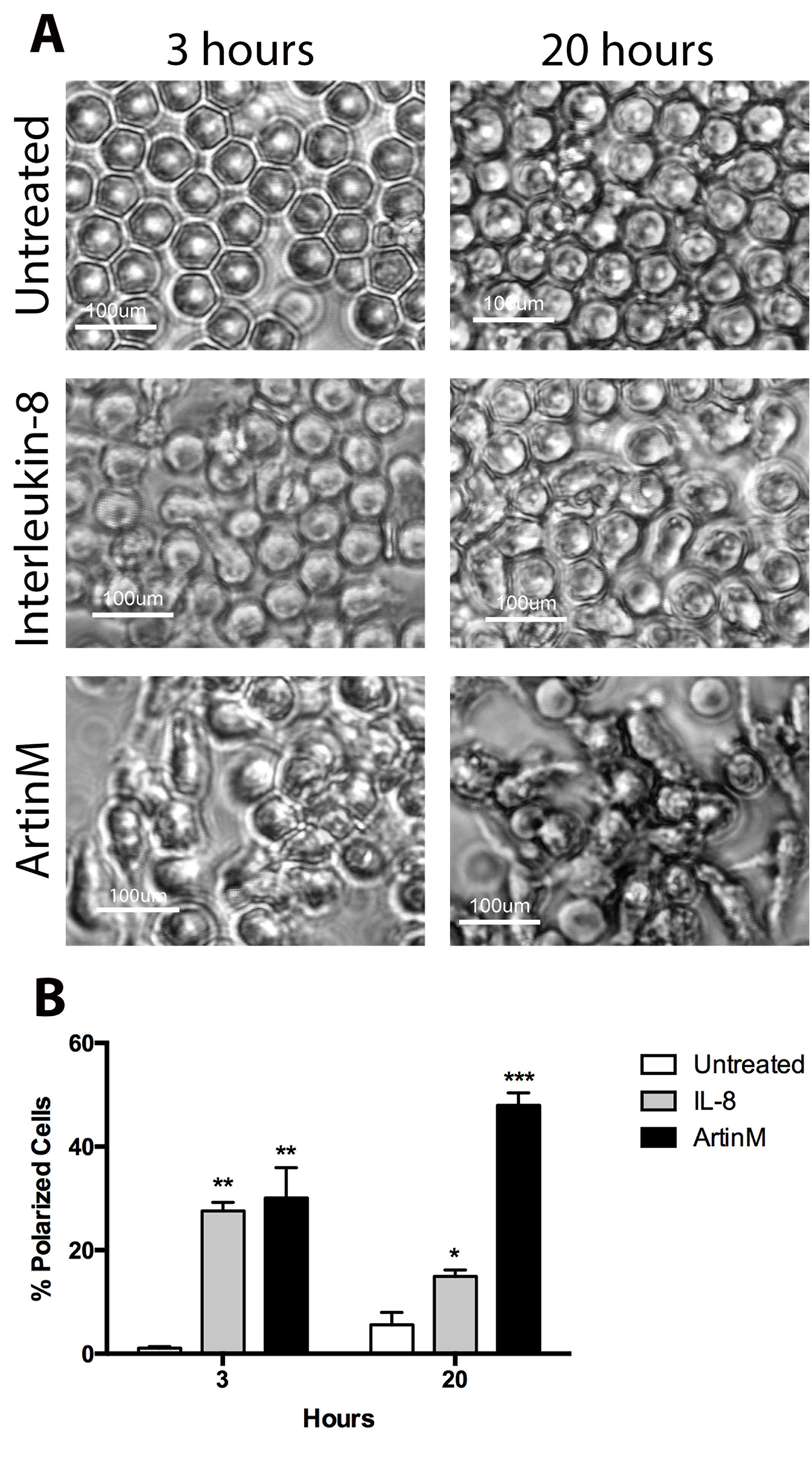

Supplement: S4 Fig — Human neutrophils were 3 and 20 h incubated with ArtinM, IL8, or medium (untreated). A—Images from the wells of the plates were obtained by inverted light microscopy coupled with an image capturing system. B–The percentage of polarized cells was determined by using the ImageJ software. Data are expressed as mean of percentage of polarized cells analyzed in 3 different fields ± SD. * p<0.05: **p<0,01: *** p<0.001 in comparison to untreated cells at the respective time. Two way ANOVA, followed by Bonferroni's post-test. (TIF) [file pntd.0004609.s004.tif]

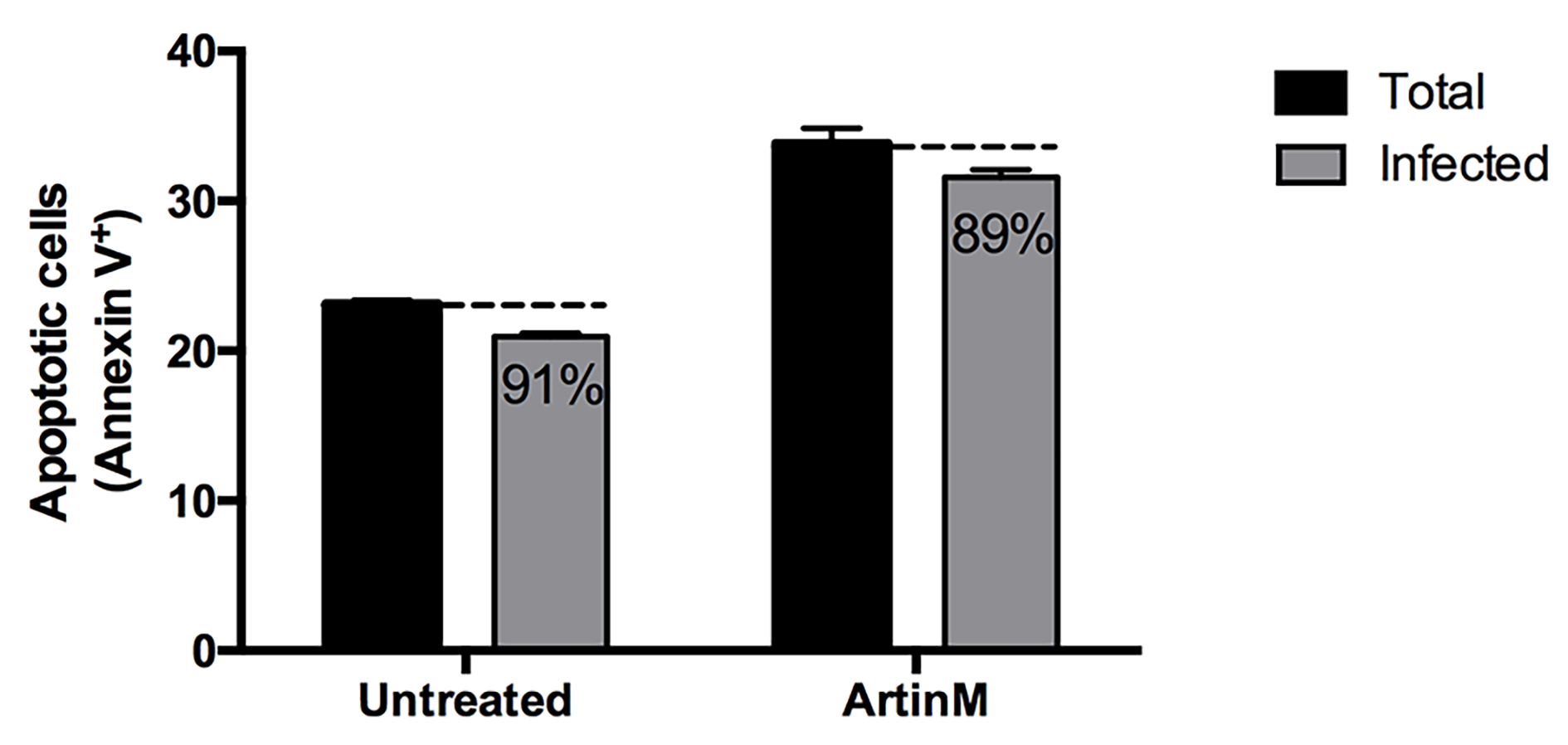

Supplement: S5 Fig — Flow cytometry analysis showed that about 90% of cells were double labelled, regardless the ArtinM treatment. (TIF) [file pntd.0004609.s005.tif]
